# Murine Leukemia Virus Spreading in Mice Impaired in the Biogenesis of Secretory Lysosomes and Ca^2+^-Regulated Exocytosis

**DOI:** 10.1371/journal.pone.0002713

**Published:** 2008-07-16

**Authors:** Wai-Tsing Chan, Nathan M. Sherer, Pradeep D. Uchil, Edward K. Novak, Richard T. Swank, Walther Mothes

**Affiliations:** 1 Section of Microbial Pathogenesis, Yale University School of Medicine, New Haven, Connecticut, United States of America; 2 Department of Molecular and Cellular Biology, Roswell Park Cancer Institute, Buffalo, New York, United States of America; The Research Institute for Children at Children's Hospital New Orleans, United States of America

## Abstract

**Background:**

Retroviruses have been observed to bud intracellularly into multivesicular bodies (MVB), in addition to the plasma membrane. Release from MVB is thought to occur by Ca^2+^-regulated fusion with the plasma membrane.

**Principal Findings:**

To address the role of the MVB pathway in replication of the murine leukemia virus (MLV) we took advantage of mouse models for the Hermansky-Pudlak syndrome (HPS) and Griscelli syndrome. In humans, these disorders are characterized by hypopigmentation and immunological alterations that are caused by defects in the biogenesis and trafficking of MVBs and other lysosome related organelles. Neonatal mice for these disease models lacking functional AP-3, Rab27A and BLOC factors were infected with Moloney MLV and the spread of virus into bone marrow, spleen and thymus was monitored. We found a moderate reduction in MLV infection levels in most mutant mice, which differed by less than two-fold compared to wild-type mice. *In vitro*, MLV release form bone-marrow derived macrophages was slightly enhanced. Finally, we found no evidence for a Ca^2+^-regulated release pathway *in vitro.* Furthermore, MLV replication was only moderately affected in mice lacking Synaptotagmin VII, a Ca^2+^-sensor regulating lysosome fusion with the plasma membrane.

**Conclusions:**

Given that MLV spreading in mice depends on multiple rounds of replication even moderate reduction of virus release at the cellular level would accumulate and lead to a significant effect over time. Thus our *in vivo* and *in vitro* data collectively argue against an essential role for a MVB- and secretory lysosome-mediated pathway in the egress of MLV.

## Introduction

Retroviruses such as the murine leukemia virus (MLV) and the human immunodeficiency virus (HIV) have been reported to accumulate in intracellular vesicles, called multivesicular bodies (MVB). It has been proposed that budding into MVB followed by release of the viral content could contribute to the overall amount of viral dissemination [1]–[4], in addition to the “classical” egress from the plasma membrane [5]–[8]. How retroviral particles reach late endosomal vesicles has remained a matter of debate. In one model, Gag would be sorted directly from the Golgi via a process dependent on the AP-3 and the E3 ligase Posh [9]–[11]. Env may be co-recruited to Gag via an interaction with TIP47, an effector of the small GTPase Rab9 [12], [13]. Downregulation of TIP47 as well as Rab9 blocks HIV release [12], [14]. In contrast, compelling evidence has recently been reported that retroviral HIV Gag accumulating in MVBs originates from the plasma membrane [15]–[18]. Importantly, interference with endocytosis blocked the accumulation of HIV Gag at MVBs, yet did not interfere with the release of virus infectivity from cells [16], [17]. Consistently, a recent time-lapse microscopy study directly visualized the assembly and budding of HIV at the plasma membrane [19]. Finally, a compartment in macrophages with MVB-like features that harbors infectious HIV was found to be continuous with the plasma membrane [20], [21].

While in some cell-types Gag reaches MVB-like compartments either via the Golgi or by re-endocytosis from the plasma membrane, a critical question remains as to whether viral particles accumulating in intracellular compartments can later be released by fusion with the plasma membrane. Release may represent a regulated event, and much excitement for a potential MVB pathway has originated from the idea that cell-cell contact could trigger MVB mobilization and polarized release [2], [3], [22]. Particularly for antigen presenting cells such as dendritic cells and macrophages, it has been proposed that interactions with T-cells can mobilize MVBs to transport HIV directly to cell-cell contact sites [22]–[24]. One way to induce the secretion of MVBs and lysosomes is through Ca^2+^ signaling. Treatment of HIV-infected cells with Ca^2+^ ionophores has been shown to dramatically increase virus release although the mechanism is not well understood [9], [25].

There is substantial *in vitro* evidence that retroviruses can accumulate within the lumen of multivesicular bodies (MVB), but the relevance of an MVB release pathway *in vivo* has not been tested. Here, we take advantage of several mouse model of the Hermansky-Pudlak syndrome (HPS) and Griscelli syndrome (GS), human genetic disorders characterized by hypopigmentation and immunological defects [26]–[28]. These defects are caused by a set of autosomal recessive mutations that affect the proper biogenesis or trafficking of MVBs and other lysosome-related organelles (LROs), including melanosomes, platelet dense granules, and lytic granules. The corresponding mice exhibit a light coat-color due to defects in melanosome biogenesis causing defects in pigmentation. In addition, prolonged bleeding times due to defects in the release of platelet dense granules are also observed. In mice, HPS is caused by a diverse set of 15 genetic mutations, 10 of which can be categorized into three distinct complexes called Biogenesis of Lysosome-related Organelle Complexes (BLOC-1, 2, or 3) [28], [29]. Defects in these complexes lead to aberrations in the synthesis of LROs. 4 of the remaining 5 mutations are known to affect the trafficking of vesicles to and from LROs [28]. Two of these mutations (*pearl and mocha*) affect subunits of the AP-3 complex, disrupting the trans-Golgi sorting of membrane proteins to endosomes and leading to an increased presence of lysosomal proteins (CD63, Lamp-1) at the plasma membrane [30], [31]. Cytotoxic lymphocytes of AP-3 mutants are also unable to destroy target cells, due to the inability of secretory granules to migrate along microtubules and polarize towards immunological synapses [32], [33].

In addition to HPS mutants, mutations in the gene encoding Rab27a cause an autosomal recessive disease in humans called Griscelli syndrome (GS) that is also characterized by hypopigmentation and immunological defects [34], [35]. Unlike HPS mutants, Rab27a mutant mice (*ashen*) generate normal secretory granules, but these compartments are unable to fuse with the plasma membrane during exocytosis [27], [36], [37]. In dendritic cells, Rab27a is also required for fusion of secretory lysosomes with phagosomes [38].

Because mouse models of the Hermansky-Pudlak syndrome and the Griscelli syndrome exhibit general defects in MVB and lysosome biogenesis, we have tested the ability of MLV to spread in these mice. MLV replication was found to be only moderately affected in mutant mice arguing against an essential role of these genes in virus replication. Furthermore, visual and biochemical analyses reveal no effect of Ca^2+^ signaling on the kinetics of MLV assembly and release. Collectively, these data argue against an important role for MVB biogenesis and egress in the productive replication and spread of MLV.

## Results

### MLV spreads in mouse models for the Hermansky-Pudlak syndrome (HPS) and Griscelli syndrome (GS)

**Figure 1:**
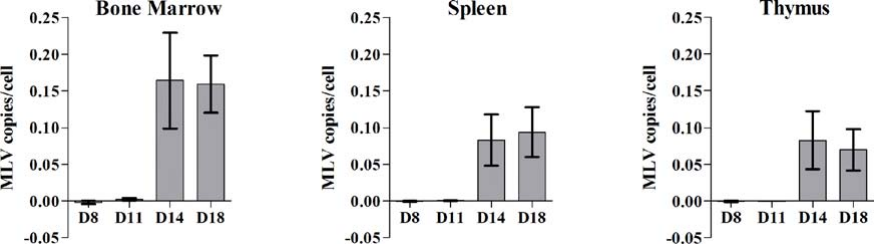
Detection of MLV infection in wildtype C57BL/6 mice. C57BL/6 mice were infected with MoMLV by intraperitoneal injection 3 days after birth and sacrificed at indicated days after infection. Genomic DNA from each organ was independently isolated and real-time PCR was performed to quantify the number of integrated proviral MLV genomes. MLV was first detected by day 11, and mice became viremic by day 14. Day 18 was selected as a suitable timepoint to sacrifice all mice for each *in vivo* experiment.

To test the contribution of a MVB pathway to the replication of MLV *in vivo*, wildtype and mutant mice defective in melanosome biogenesis were analyzed for their ability to support MLV infection [26]. Mice lacking Rab27a (*ashen*) were compared to the background strain *C3H/HeJ*. All other mice, lacking functional AP-3 (*pearl*), BLOC-1 (*pallid*), BLOC-2 (*ruby-eye*), BLOC-3 (*light-ear*), BLOC-2,3 (*cocoa/light-ear*), and the triple knockout BLOC-1,2,3 (*pallid/cocoa/light-ear*) were compared to their background *C57BL6* strain. Mice were infected with 3×10ˆ3 infectious MoMLV virions intraperitoneally 3 days after birth to bypass a strong humoral antibody response that can completely control MLV infection in adult mice [39]. At different time points post-infection, the mice were sacrificed, bone marrow, spleen, and thymus were removed, and genomic DNA was isolated and analyzed by real-time PCR to quantify the number of MLV genomes. This copy number was normalized to the number of copies of host actin-A1 in each sample and subsequently to the number of cells. Viral replication was first detected 10–14 days post-infection (Figure 1). Bone-marrow, spleen and thymus became infected simultaneously, differing from reports published for a MLV variant that initially targets the bone-marrow [40]. Infection levels were highly reproducible beyond day 14, and day 18 was chosen for an end point analysis of MLV replication levels.

**Figure 2:**
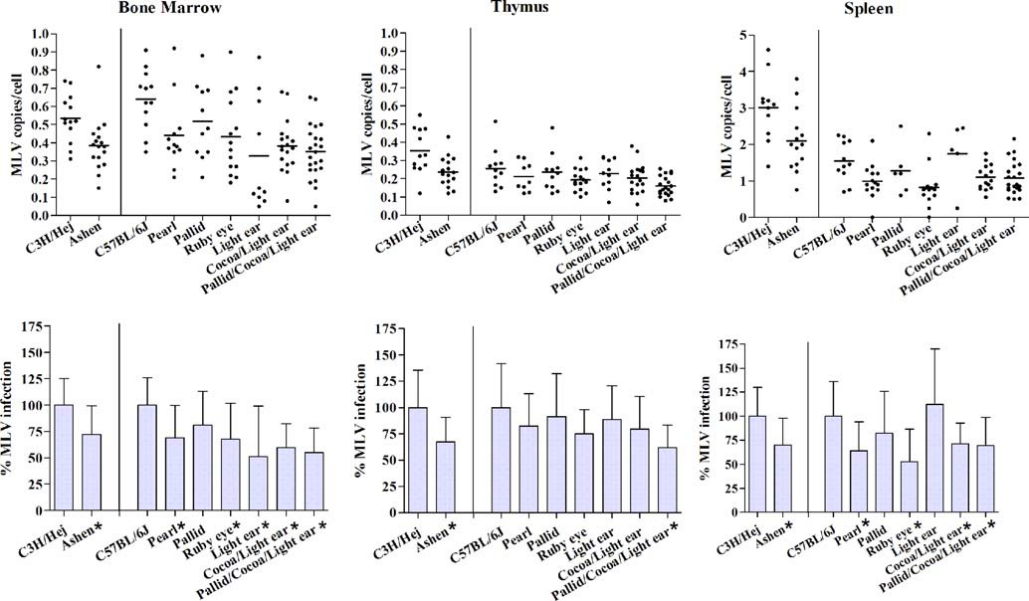
MLV spreading in mouse models for Hermansky-Pudlak and Griscelli syndromes. Wildtype and mutant mice were infected with Moloney MLV by intraperitoneal injection 3 days after birth. Bone marrow, spleen, and thymus were harvested 18 days post-infection and the genomic DNA analyzed. The copy number for proviral MLV was normalized to cell numbers using internal standards for actin A1. Rab27a (*ashen*) is compared to its wildtype background *C3H/Hej*, while the remaining AP-3 (*pearl*), BLOC-1 (*pallid*), BLOC-2 (*ruby-eye*), BLOC-3 (*light-ear*), double mutant BLOC-2,3 (*cocoa/light-ear*) and triple mutant (*pallid/cocoa/light-ear*) are compared to *C57BL/6J*. The statistical analysis with standard deviations is presented in the lower panel, in which infection levels observed in both wild-type background mice are set to 100%. Values labeled with an asterisks (*) indicates statistically significant differences to wildtype using the non-parametric Mann-Whitney double T-Test with a confidence level of 95%.

For all mutant mice tested, a moderate up to two-fold % decrease in viral load was seen in all three organs compared to wildtype (Figure 2). In some cases, this moderate reduction was found to be statistically significant by non-parametric Mann-Whitney double T-Test with a confidence level of 95% (indicated by * in Figure 2).

### MLV is efficiently released from primary macrophages derived from HPS mice

**Figure 3:**
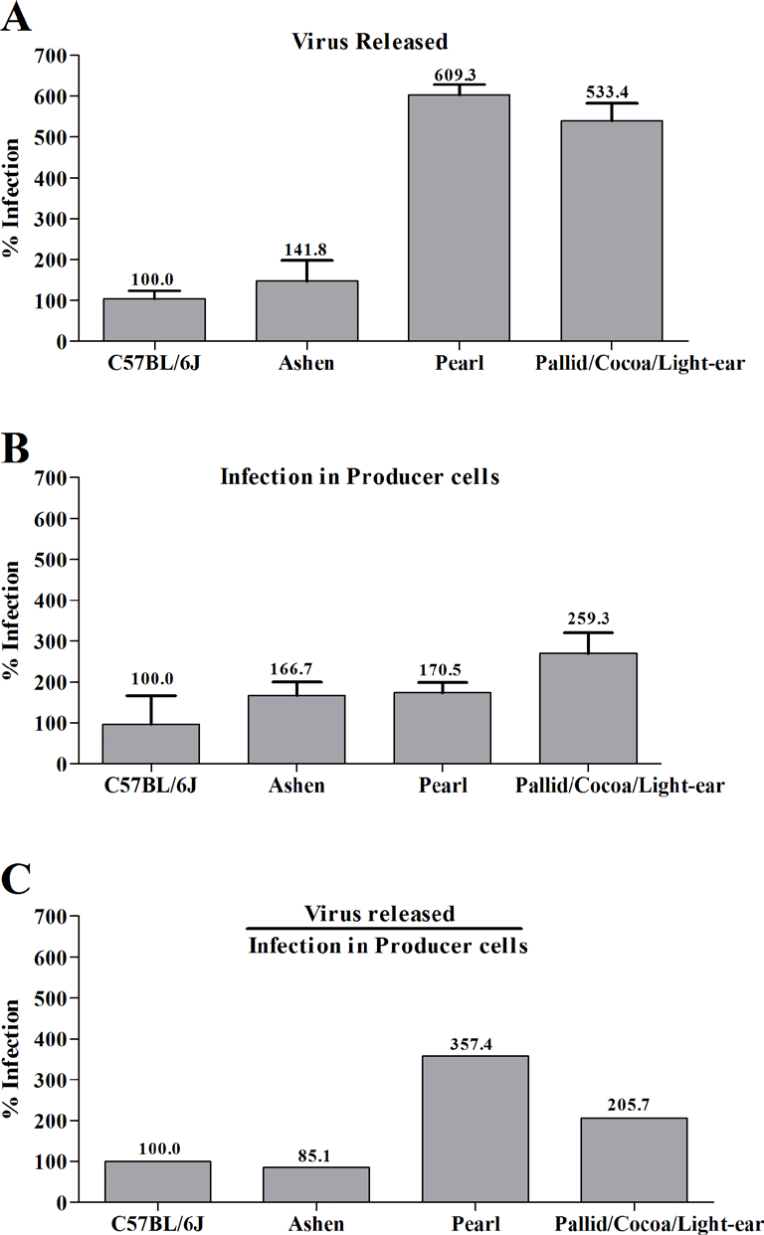
MLV release from bone marrow derived macrophages is enhanced in HPS mutants. (A) Percentage release of MoMLV into the supernatant by chronically infected macrophages relative to wildtype. Macrophages from respective mice were chronically infected for 7 days, washed extensively with PBS; and supernatants were harvested after 48 hours and titered on DFJ8 cells. Genomic DNA from DFJ8 cells was extracted, and proviral insertions were quantified by real-time PCR. (B) Percentage infection of macrophages by MoMLV. Genomic DNA from chronically infected macrophages previously described were extracted and quantified by real-time PCR. (C) The amount of MoMLV released in the supernatant of macrophages was normalized to the relative amount of infection of producer macrophages. Error bars represent standard deviations from n = 6 independent experiments. The levels of infection observed for *C57BL/6J* were set to 100%.

**Figure 4:**
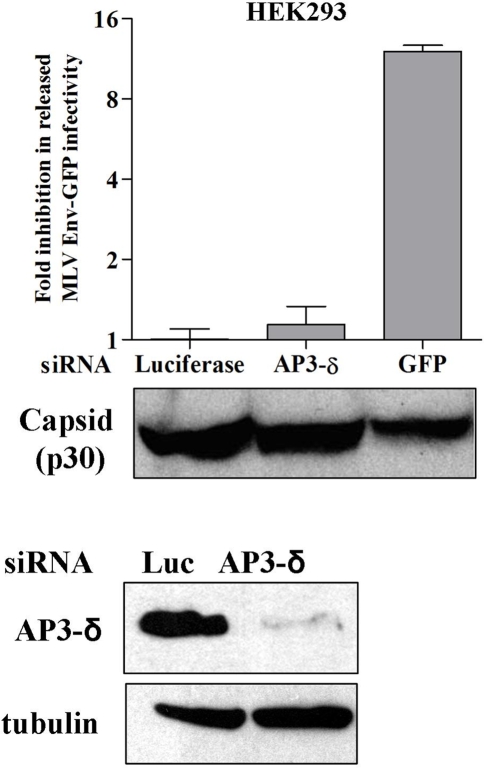
Silencing of AP-3δ subunit does not effect MLV release. (A) HEK293 cells were transfected with siRNA targeting luciferase (negative control), AP-3δ subunit or GFP (positive control). 24 h later these cells were transfected with plasmids encoding full-length MLV expressing Env-GFP. 48 h later the infectivity as well as p30 MLV capsid released into the supernatant was determined. While targeting AP-3 has no effect on MLV release, targeting the GFP within the Env and full-length genome messages results in the inhibition of virus release. (B) Western blot of HEK293 cells lysates treated with AP-3δ or luciferase siRNA using AP-3δ and tubulin (loading control) specific antibodies.

In order to determine the effects of HPS factors in virus release on a cellular level, bone-marrow derived macrophages were isolated from wildtype and mutant mice and tested for their ability to release virus (Figure 3A, B). Although the level of infection in the macrophages from all mice varied slightly, none of the three mutants displayed a decrease in viral output compared to wildtype. In fact, after normalizing the amount of virus released to the level of infection in the corresponding producer cells, macrophages from the pearl (AP-3) and triple knockout BLOC-1,2,3 mutants released two to four fold more virus (Figure 3C). Moreover, when the δ subunit of AP-3 was downregulated using siRNA in HEK 293 cells with efficiency as high as 95%, released MLV infectivity and capsid were unchanged (Figure 4). These results demonstrate that the mutation of HPS factors and the absence of functional AP-3 does not hinder viral egress, but in some cell-types actually enhances release.

### Ca^2+^-regulated release of secretory lysosomes does not significantly contribute to MLV release *in vitro* and *in vivo*

**Figure 5:**
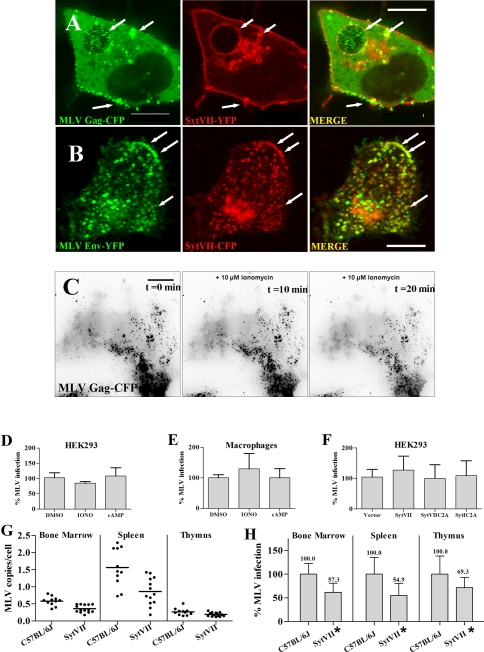
MLV release is not mediated by secretory lysosomes in a Ca^2^ dependent manner. A A confocal zslice through the center of a HEK293 cell showing MLV GagCFP particles green accumulated within the interior of a swollen Syt VIIYFP positive vesicle red under conditions of infectious virus production. White arrows indicate areas of colocalization. B An experiment as in A showing MLV EnvYFP green localized to peripheral vesicles white arrows positive for Syt VIICFP red. Image is a 3D reconstruction of a confocal zstack. C Individual frames from a timelapse movie by TIRFM of a 293 cell generating GagCFPlabeled MLV and treated with 10 M ionomycin. Images indicate pretreatment left panel, 10min middle panel and 20min right panel after ionomycin treatment. Size bar corresponds to 10 m. D Viral titers of supernatants harvested from 293 cells generating *LacZ*encoding MLV after incubation for 60min in normal growth media, media containing 10 M ionomycin, and media containing 1mM dibutyryladenosine cAMP. E Chronically infected mouse macrophages from C57BL6, seven days postisolation, were washed extensively and incubated for 30min in media, media containing 10 M ionomycin, or media containing 1mM dibutyryladenosine cAMP. Supernatants were harvested and titered on DFJ8 cells prior to quantitative realtime PCR to detect proviral insertions. F Transfection was performed as for C in the additional presence of 25ng of parental plasmid vector alone or plasmids encoding YFPtagged fulllength, dominant negative C2A domain of Syt VII or control Syt I, respectively. G Wildtype C57BL6J B6 and Syt VII mutant mice infected by intraperitoneal injection with Moloney MLV 3 days after birth. Realtime PCR was performed on genomic DNA extracted from indicated organs to detect MLV insertions as previously described.* denotes statistically significant differences from wildtype by nonparametric MannWhitney double TTest with a confidence level of 95. H Percentages of G for each organ.

Secretion of MVBs and other secretory lysosomes is regulated by Ca^2+^ in a process that is controlled by Synaptotagmin VII (Syt VII) [41], [42]. To test for a potential role for Syt VII in MLV release, we asked if MLV Gag would co-localize to Syt VII-positive vesicles. MLV assembly was visualized in HEK 293 cells using fusion proteins to MLV Gag and Env as previously described [3]. When Syt VII-YFP was co-expressed, a co-localization particularly with peripheral lysosomal vesicles [43] was observed (Figure 5A, B). Because Syt VII-YFP like the endogenous protein localizes to CD63 containing vesicles [43], [44], this co-localization raised the question if MLV is released via Syt VII-dependent secretion of lysosomes.

Secretion of lysosome related organelles is stimulated in response to a rise in intracellular Ca^2+^ and can be induced by Ca^2+^ ionophores and cAMP [41], [45]. Two previous studies have reported increased HIV particle release in response to ionomycin treatments [9], [25]. We predicted that Ca^2+^ driven MVB fusion with the plasma membrane would lead to the increased exposure of viral antigens at the cell surface as well as the increased secretion of intralumenal virus like particles into the culture supernatant. To this end we monitored MLV Gag-GFP distributions in single cells using live time lapse wide-field fluorescence imaging. The addition of 10 μM ionomycin demonstrated no effect on Gag-GFP delivery to the cell surface from intracellular sources (data not shown). In addition we applied total-internal reflection fluorescence microscopy (TIR-FM) to detect the possible fusion of endolysosomal vesicles carrying viruses with the plasma membrane. TIR-FM utilizes evanescent waves that are produced when light is totally reflected at the glass-water interface. Because these waves decay exponentially from the interface, they penetrate only to a depth of approximately 100 nm into the sample medium. Thus TIR-FM can be used to fluorescently excite the dorsal face of the cell to ideally monitor events occurring specifically at or near the plasma membrane. Using TIR-FM, we found no evidence of Gag-positive MVB fusion with the cell surface in response to ionomycin (Figure 5C).

In parallel, we harvested the culture supernatants after ionomycin and cAMP treatment of HEK293 cells producing a Lac-Z encoding MLV variant or primary macrophages producing infectious wildtype MLV. In both experiments, we observed no significant difference in virus or virus-like particle release between treated and untreated cells (Figure 5D, E). Moreover, the expression of the dominant-negative C2A domain of Syt VII had no effect on MLV release from HEK 293 cells (Figure 5F). Finally, to directly test a potential role for Syt VII in viral release, we tested MLV spread in Syt VII knockout mice [46]. MLV spread was moderately reduced in mice lacking Syt VII, a result deemed significant by a non-parametric Whitney-Mann double T-test (Figure 5G, H).

## Discussion

In this study, we have conducted *in vivo*, *ex vivo*, and *in vitro* experiments that collectively argue that a MVB-mediated pathway of viral egress does not play an essential role in the dissemination of MLV. *In vivo*, BLOC-1,2,3, AP-3, Rab27a, and Syt VII mutant mice sacrificed at 18 days post-infection exhibited only a moderate decrease in viral load in the bone marrow, spleen, and thymus when compared to wildtype mice. At cellular level, MLV release from infected macrophages lacking AP-3, Rab27a, or BLOC-1,2,3 was not inhibited, but rather enhanced when compared to wildtype. These data suggest that the Golgi-endosomal sorting pathway plays an inhibitory role in MLV release. Finally, we demonstrated *in vivo* and *in vitro* that the fusion of secretory lysosomes with the plasma membrane in response to elevated calcium levels do not increase the level of MLV release.

In recent years, a number of groups have demonstrated that both HIV and MLV have the ability to accumulate and bud intracellularly into the lumen of MVB *in vitro*. It had been speculated that viruses accumulating within this compartment may contribute to the overall viral dissemination through a MVB-mediated pathway of virus release, similar to the release of exosomes, small 50–200 nm vesicles that accumulate in MVBs and are released into the culture supernatant [4], [47]. However, our studies on MLV spread *in vivo* using mouse models of the Hermansky-Pudlak syndrome does not support an important role for an MVB-mediated release pathway. These mutant mice were originally identified based on changes in the coat-color indicating defects in the biogenesis of melanosomes, a specialized MVB found in keratinocytes that carries pigment [48]. At least in the case of *pearl* (impaired in AP-3) and *ashen* mice (impaired in Rab27a), a general defect in the biogenesis and release of secretory lysosomes has been observed [32], [33], [36], [37]. However, both mice became infected with MLV infection levels similar to wild-type or just moderately reduced. When one considers that it takes two weeks before MLV infection spreads into all organs, implying many consecutive rounds of infection, an essential role of these factors in virus dissemination is unlikely. *In vivo*, even moderate effects at the single cell level should accumulate over time into a sizeable phenotype. In contrast, *in vitro* cultured primary macrophages showed enhanced virus release. MLV spreading was also not affected in mice lacking BLOC components. Recent data suggest that BLOC mice may not exhibit general defects in the biogenesis of secretory lysosomes, but rather play a specific role in the biogenesis of melanosomes [49].

While our data argue against an important role of MVBs in MLV release, they cannot exclude the possibility that this pathway contributes to virus release in specific cell types. In our study, newborn mice were infected that lack a fully developed immune system and therefore lack fully differentiated antigen presenting cells. Hence, we cannot exclude the possibility that MLV uses an MVB pathway during the infection of adult mice, in which the disease is quickly controlled by an antibody mediated response [39].

In contrast to MLV, HIV may potentially use a dissemination pathway involving MVB and secretory lysosomes [9], [25]. Functional AP-3 was reported to be critical for the sorting and release of HIV Gag from cells suggesting that trafficking of HIV Gag from the Golgi towards late endosomes/MVBs is necessary for HIV release [10]. However, recent reports provide evidence that the accumulation of HIV in late endosomal membranes is due to endocytosis of HIV virions budding at the plasma membrane and did not lead to a release of infectious virions [16], [17], [19] questioning a role of MVB in HIV release. In fact, these MVB-like compartments filled with infectious HIV as observed in macrophages are continuous with the plasma membrane [20], [21].

Finally, ionomycin treatment has been shown to stimulate HIV release [9], [25]. However, in the case of MLV, we did not detect any increases in virus release under such conditions. To further study this *in vivo*, we utilized the Syt VII knock out mouse model. Syt VII is a membrane protein found on lysosomal membranes known to regulate lysosomal exocytosis and the fusion of secretory granules with the plasma membrane through calcium signaling [42], [46], [50]. Although we demonstrate that Syt VII colocalizes with both MLV Gag and Env, we could not see a strong block to the spread of MLV in Syt VII knockout mice or in cells expressing dominant negative Syt VII C2A. Thus, our data collectively argue against a functional role for MVB- and secretory lysosome-mediated pathway in the egress of MLV in neonatal mouse model.

## Materials and Methods

### Mice:

Pearl, pallid (*pa*), ruby-eye (*ru*), light-ear (*le*), cocoa/light-ear (*co/le*), pallid/cocoa/light-ear (*pa/co/le*), ashen (*ash*) were kindly provided by Richard Swank (Roswell Park Cancer Institute, Buffalo, NY) [26], [28]. Wildtype *C57BL/6* and *C3H/Hej* mice were from JAX (Jackson Laboratory, Maine). Synaptotagmin VII (*sytVII*) mice [46] were kindly provided by Norma Andrews (Yale University, New Haven, CT). Mice were handled according to the institutional guidelines for animal husbandry and experiments.

### *In Vivo* Mouse Experiments:

Wildtype and mutant mice were infected with 20 μl Moloney MLV (MoMLV) stock [51] by intraperitoneal injection 3 days after birth. When titered on susceptible DFJ8 cells [52], 20 μl corresponded to 3×10^3^ integrated MLV genomes. A 10 fold lower amount of virus resulted in the infection of about half of the mice demonstrating that this amount of virus was required to reproducibly infect all mice in a study group. Mice were sacrificed 18 days post-infection, and bone marrow, spleen, and thymus were removed and homogenized. Genomic DNA was isolated by Qiagen DNAeasy from each organ separately, and proviral integrations quantified by real-time PCR (SYBR-GREEN; Bio-Rad, Hercules, CA) using primers for MLV LTR [53] and parallel standards for cytochrome b and ActinA1. The copies of MLV proviral genomes were normalized to the number of ActinA1 copies per cell. Towards this end, the number of cells was determined prior to DNA preparation and RT-PCR. To test the significance of our results, we performed a non-parametric Whitney-Mann double T-test using Graph Prism version 4.00.

### Macrophage Experiments:

Primary mouse macrophages were generated by isolating the bone marrow from femurs of wild-type and mutant mice. The resulting cells were then plated onto non-tissue-culture treated 6-well plates at a density of 10^6^ cells per well in macrophage culture medium containing RPMI 1640 medium supplemented with 20% L-cell supernatant and 10% FBS. After two days, the macrophages were infected with MoMLV in the presence of 5 μg/ml polybrene at a MOI of 1. At day 7 post-infection, macrophages were washed extensively five times with phosphate buffered saline. Macrophages were then incubated in 1 ml macrophage culture media and supernatants collected after 48 hours. Genomic DNA was then isolated from the producer macrophages, and the resulting supernatants were used to infect DFJ8 cells. After 2 days, genomic DNA from the infected DFJ8 cells was isolated. The number of copies of integrated MLV and cellular actinA1 from genomic DNAs of both producer macrophages and corresponding target DFJ8 cells were then quantified by real-time PCR as described above.

### Budding Assays:

1×10^6^ HEK 293 cells per 35 mm dish were transfected using FuGene 6 as previously described [3] with 400 ng each of MLV Gag-Pol, Env, and LTR-LacZ with or without 50 ng of plasmids encoding full-length Syt VII, dominant-negative Syt VII C2A domain, control Syt I C2A domain [42]. At 24 hours post-transfection, cells were washed, trypsinized, and diluted two-fold in fresh media before replating. Culture supernatants containing virus were collected 24 hours later (48 hours post-transfection), filtered and titered by serial dilution on DFJ8 cells. Alternatively, HEK 293 cells, transfected as above, were washed five times and incubated in the appropriate growth media containing ionomycin or di-butyryl adenosine cAMP (Sigma, St. Louis, MO) for 1 hour. To reduce the effects of entry on virus titration, culture supernatants were either diluted 1:10 in normal growth media or viruses were sedimented through a 15% sucrose cushion by centrifugation at >20,000×g for 2 h prior to resuspension in 1 ml media. In the AP-3 silencing experiment, 5×10^5^ HEK293 cells in 48 well plates were transfected with 80 nM siRNA (Samchully Pharm. Ltd., Seoul, Korea) specific to AP-3δ subunit, GFP (positive control) or luciferase (negative control) using Lipofectamine 2000 (Invitrogen, CA). 24 hours post transfection cells were split 1:4 into 4 wells of a 48 well plate and transfected again as above with 100 ng plasmid encoding full-length MLV genome carrying a GFP insertion into the envelope protein. After an additional 48 h, the culture supernatants were harvested and applied onto DFJ8 for MLV titration. DFJ8 cells were harvested 48 h after infection and analyzed by FACS to determine the number of GFP-positive cells. Fold inhibition in release was calculated using a ratio of the percent GFP positive cells from experimental samples transfected with AP-3δ-specific or GFP-specific siRNA and those transfected with luciferase control siRNA. Downregulation of AP-3δ was tested by western blot using antibodies to δ subunit of AP-3 (120 kDa; BD transduction laboratories). The sense strand siRNA sequence used for targeting AP-3 was 5′ UCUGCAAGCUGACGUAUUUdTdT-3′, GFP was 5′-CGGCCACAAGUUCAGCGUGUCdTdT-3′ and luciferase was 5′-CUUACGCUGAGUACUUCGAAAdTdT-3′.

### Live imaging and fluorescence microscopy:

Fluorescence microscopy was carried out as previously described [3], [54] using the 100×objective of a LSM510 confocal microscope equipped with a Zeiss Axiovert 100 M base (Zeiss Microimaging, Jena, Germany). Live cell imaging using total internal reflection fluorescence microscopy was as previously described [55] using the 60×(1.45 NA) oil-immersion objective of a modified Olympus IX-70 microscope. Drugs were added during imaging by 1:10 dilution into the DMEM media to achieve the appropriate final concentration. Synaptotagmin VII-YFP was described previously [44].
